# Malaria care-seeking and treatment ideation among gold miners in Guyana

**DOI:** 10.1186/s12936-022-04045-5

**Published:** 2022-01-31

**Authors:** Bolanle Olapeju, Camille Adams, Sean Wilson, Joann Simpson, Gabrielle C. Hunter, TrishAnn Davis, Lyndsey Mitchum, Horace Cox, Kashana James, Jennifer Orkis, J. Douglas Storey

**Affiliations:** 1 Johns Hopkins Center for Communication Programs, 111 Market Place, Suite 310, Baltimore, MD 21202 USA; 2 Department of Health, Behavior and Society, Johns Hopkins Bloomberg School of Public Health, 624 N. Broadway, Baltimore, MD 21231 USA; 3 Breakthrough ACTION Guyana, Georgetown, Demerara-Mahaica Guyana; 4 National Malaria Programme, Ministry of Health, Georgetown, Demerara-Mahaica Guyana

**Keywords:** Malaria, Guyana, Care-seeking, Treatment, Ideation, Behaviour

## Abstract

**Background:**

Although miners are a priority population in malaria elimination in Guyana, scant literature exists on the drivers of malaria-related behaviour. This study explores the relationship between gold miners’ malaria-related ideation and the adoption of malaria care-seeking and treatment behaviours including prompt care-seeking, malaria testing, and self-medication.

**Methods:**

Data are from a cross-sectional quantitative survey of 1685 adult miners between the ages of 18–59 years who live in mining camps in Regions 1, 7, and 8. The analysis focused on miners who reported an episode of fever in the past year (n = 745). Malaria care-seeking and treatment ideation was defined as a composite additive score consisting of the following variables: general malaria knowledge, perceived severity, perceived susceptibility, beliefs, perceived self-efficacy, perceived norms, interpersonal communication, and perceived response efficacy. Multivariable logistic regressions explored the relationship between ideation on care-seeking/treatment behaviours, controlling for confounding variables.

**Results:**

Most miners with a recent episode of fever had perceived risk (92%), self-efficacy (67%), susceptibility (53%) and high malaria knowledge (53%). Overall, miners' care-seeking/treatment ideation score ranged from 0 to 8 with a mean of 4.1. Ideation scores were associated with higher odds of care-seeking for fever (aOR: 1.19; 95% CI 1.04–1.36), getting tested for malaria (aOR: 1.22; 95% CI 1.07–1.38) and lower odds of self-medication (aOR: 0.87; 95% CI 0.77–0.99).

**Conclusions:**

A national community case management initiative is using study findings as part of its scale-up, using volunteers to make testing and treatment services more accessible to miners. This is complemented by a multi-channel mass media campaign to improve miners’ ideation. Communication messages focus on increasing miners’ knowledge of malaria transmission and symptoms, encourage positive beliefs about malaria testing and volunteer testers, promote evidence about the effectiveness of testing, and reminders of how quick and easy it is to get a malaria test with the community case management initiative. Study findings also have implications for efforts to eliminate malaria across the Guiana Shield.

## Background

According to the 2020 World Malaria Report, Guyana had over 20,000 cases of malaria in 2019, an increase in case incidence of more than 40% compared with 2015 [1]. Of the 782,775 total population of Guyana, 85,432 (9%) are considered at high risk of malaria [1]. In addition, the hinterland regions 1, 7, 8, and 9 have the country’s highest malaria transmission rates [2]. Malaria burden is particularly high among remote and mobile gold mining populations, and evidence suggests that malaria surges are driven by rising gold prices and increased gold mining activities in areas that are prime breeding environments for mosquitoes [3]. In addition, migrant and mobile mining populations are hard to reach with information and services [4].

In Guyana, multiple *Plasmodium* species are responsible for malaria transmission; over 60% of the local cases are due to *Plasmodium vivax*, while *Plasmodium falciparum* and mixed species cause the rest [1, 5]. The first-line treatment for uncomplicated falciparum malaria in Guyana is a three-day course of artemether-lumefantrine and primaquine, while chloroquine and primaquine taken over 14 days are the treatment for vivax malaria [1]. These treatments are recommended upon diagnosis of malaria from either microscopy or a rapid diagnostic test (RDT) typically conducted after people with fever or other malaria symptoms seek medical care from a qualified health provider [2].

Many individuals with fever cases do not get tested or treated in Guyana. Recent data published shows that in the hinterland Regions 1, 7, and 8, less than two-thirds of gold miners with fever sought care for their fever from a health facility or got tested for malaria. On the other hand, about one-half of gold miners with fever self-medicated before or instead of seeking care [6, 7]. The high prevalence of the disease in this hard-to-reach population, in conjunction with difficult access to and incorrect use of anti-malarial treatments, could favour the emergence of resistant parasites [8]. Although miners are a priority population for malaria elimination in Guyana, scant literature exists on the drivers of reported malaria-related behaviour, including the role of structural factors such as poverty, migration, and limited access to health facilities [8].

Little information is available on psychosocial factors influencing malaria care-seeking and treatment among miners in Guyana. In general, research has proven the influence of knowledge, perceptions, decision-making patterns, and interpersonal relationships on health care-seeking [9–12]. The conceptual framework of the study draws from the ideation model, a predictive model of behaviour change that focuses on the multiple, inter-related psychosocial variables that commonly influence individual behaviour [13–15]. The ideation model recognizes that most behavioural decisions are driven by multiple psychosocial factors, often simultaneously. The ideation model has three components, each of which comprises several elements: (1) cognitive elements which include variables such as attitudes, beliefs, values, perceived risk, subjective norms, and self‐image; (2) emotional elements which include emotional response, empathy, and self‐efficacy variables; and (3) social elements including social support and influence, spousal communication, and personal advocacy variables [16]. These variables function like risk-factors for disease, but in a positive way: the more of these ideational variables that apply to a person, the more likely that individual is to adopt the behaviour. These ideational variables are also influenced by communication, whether through social interaction, mass media, or interpersonal communication. The factors work both individually and synergistically to influence health outcomes. Research has demonstrated the relationship between ideation and malaria behaviour, including insecticide-treated net use [17], intermittent preventive treatment of malaria in pregnancy [18], and care-seeking for children under five [19]. Not much is known about the association between ideation and care-seeking among adults, particularly in the Guyana context. A study in Regions 1, 4, 6, and 8 of Guyana demonstrated the link between perceived risk and prevention behaviour for malaria and other vector-borne diseases, but did not explore other ideational variables [20].

The aim of this study is to understand how gold miners’ malaria-related ideation influences the adoption of malaria care-seeking/treatment behaviours including prompt care-seeking, malaria testing, and self-medication. Study findings will be used to inform the design of relevant social and behaviour change (SBC) interventions among this high-risk population as well as the ongoing Community Case Management Initiative (CCM) which is a volunteer testing program implemented by the National Malaria Programme (NMP) to support greater access to malaria services by miners in Regions 1, 7, 8, and 9. The programme CCM volunteer testing focuses on recruitment and training residents living and working in the communities. These volunteers are trained to perform malaria RDTs on non-pregnant adults with symptoms and provide treatment for uncomplicated cases of malaria. This study is also relevant to malaria elimination in the Guiana Shield, which includes Venezuela, Guyana, Suriname, French Guiana, and parts of Brazil and Colombia.

## Methods

### Study design and population

Full details of the research methodology have been published elsewhere [6]. In summary, the study conducted a cross-sectional quantitative survey of 1685 adult miners between the ages of 18–59 years living in mining camps in Regions 1, 7, and 8 between November and December 2019, as part of a baseline assessment for the USAID-funded Breakthrough ACTION project in collaboration with the NMP of the Guyana Ministry of Health. The baseline assessment informed the design of appropriate SBC interventions to complement the volunteer tester program and improve malaria outcomes in these regions. Prior to data collection, a total of 4051 miners were verified across Regions 1 (795 miners), 7 (2486 miners), and 8 (770 miners). The study stratified camps by region and size: large camps were defined as those with 23 miners or more, medium camps had 8–22 miners, while small camps had 7 or fewer miners. From the listings, the study drew a multistage sample of 1685 miners from 233 mining camps in the three regions, proportional to the population of gold miners as well as the size of the camps.

### Ethical considerations

Institutional review boards from the Johns Hopkins Bloomberg School of Public Health and the Guyana Ethical Review Committee performed ethical review and approval for this study. Prior to participating in the survey, all respondents provided written consent. The study maintained ethical principles such as privacy, autonomy, and beneficence throughout the study.

### Data collection

Researchers conducted interviews face-to-face with miners, which lasted about 20 min on average. Modules explored in the questionnaire included mining camp identification; personal information; knowledge about malaria, treatment, and testing; attitudes and behaviour; use of insecticide-treated mosquito nets; and exposure to information about malaria testing and treatment.

### Data analysis

The analysis focused on miners who reported an episode of fever in the past year (n = 745). The key behavioural outcomes of interest were all self-reported: (1) prompt care-seeking, defined as seeking of advice or treatment within 24 h after the onset of fever symptoms; (2) testing for malaria, defined as having blood taken for testing at any time during the fever; and (3) self-medication, defined as taking any medication for the fever before seeking advice or testing.

The key explanatory variable of interest was malaria care-seeking and treatment ideation, which was defined as a composite additive score consisting of the following variables: general malaria knowledge, perceived severity of malaria, perceived susceptibility to malaria infection, interpersonal communication with others about malaria, specific care-seeking/treatment beliefs, perceived self-efficacy (self-confidence) to protect oneself from malaria, perceived norms related to treatment adherence, and trust in available testing and treatment options (i.e., response efficacy). Table 1 shows the specific questions used to assess malaria care-seeking and treatment ideation across the elements of interest. Responses for each ideation variable were dichotomized to reflect high and low ideation (coded as 1 and 0 respectively). Thus, the ideation score was a composite of these 8 variables and ranged from 0 to 8 with a median of 5. Higher scores indicated more positive ideation. The Cronbach’s alpha for all the variables included in the ideation score was 0.76. The ideation score was further categorized into low ideation (less than the median) and high ideation (equal to or greater than the median) for some of the analyses.

**Table 1:** Variables used to construct the care-seeking ideation index

*Knowledge*
Have you ever heard of a disease called malaria?
What causes malaria?
What are the different types of malaria in Guyana?
What signs or symptoms would lead you to think that a person has malaria?
If you thought you had malaria, where could you go to get a free malaria test and treatment?
What are some things that people can do to stop them from getting malaria?
*Perceived severity*
If you became infected with malaria, how serious would that be for your health and well-being?
*Perceived susceptibility*
How likely do you think it is that you will become infected with malaria within the next 6 months?
*Perceived norms*
Generally, how many of your friends and co-workers stop taking their malaria medicine before the end of the treatment?
*Perceived self-efficacy*
I am confident that I can get a test for malaria within 24 h after I start to feel symptoms
I am confident that I could complete a full three-day treatment if I were diagnosed with *P. falciparum*
I am confident that I could complete a full 14-day treatment if I were diagnosed with *P. vivax*
*Perceived response efficacy*
The results of the tests given by volunteer testers are always accurate
A blood test for malaria is the only way to know if someone really has malaria
A person should only take malaria medicine if a health provider or tester says that a fever really is malaria
*Beliefs*
I don’t worry about malaria because it can be easily treated
Every case of malaria can potentially lead to death
When someone I know gets malaria, I usually expect them to completely recover in a few days
A person sick with fever should always receive a blood test to confirm that the sickness is malaria before taking malaria drugs
A person should go for a malaria test the same day they start to feel symptoms
If I think I might have malaria, I treat myself first, then go for a test if the symptoms get worse
A person should only use the Ministry of Health-approved medicine for malaria
A person with malaria should only use the treatment approved for the type of malaria you have
Malaria medicine that you buy in the market or a shop is as good as the ones approved by the government
Trained malaria testers in my area always have a supply of the approved treatments
*Interpersonal communication*
In the past month, have you talked with anyone about the best ways to prevent malaria?

Control variables for the analysis included sociodemographic factors: age in years (less than 35 versus 35 or more years old), sex (male versus female); region (Region 1, 7 or 8); education (no education, some primary, completed primary, some secondary, beyond secondary); marital status (not married versus married); religion (Christian versus non-Christian), mining experience (less than five versus five or more years); mobile phone ownership (yes or no); access to mass media channels, defined as access to either TV, radio, internet, or social media at least 4 times a week (no versus yes); and number of malaria episodes experienced in the past year (one, two, three or more).

Analytical methods included tests of association (Chi-square, t-tests and ANOVA used for exploratory analysis and group comparisons), as well as multivariable logistic regressions of the ideation score on care-seeking/treatment behaviour, controlling for region, gender, religion, age, marital status, education, mining experience, prior episode of malaria, ownership of a mobile phone, and access to mass media channels.

## Results

As shown in Table 2, the study population of miners with a fever in the past 12 months was predominantly male (86%) and Christian (82%). Less than one-half were older than 35 years (43%), married (42%) and had over five years of gold mining experience (50%). About two-thirds (67%) of miners had a prior episode of confirmed malaria in the past year. The majority of miners owned a mobile phone (71%), while almost half (48%) frequently accessed mass media channels such as radio, television, internet, or social media.

**Table 2:** Description of the study population, by region

Miner’s characteristics	Prevalence				Chi-square
	Region 1 (n = 157)	Region 7 (n = 386)	Region 8 (n = 202)	Total (n = 745)	P-value
Male	82.4	88	82.9	85.8	0.260
Christian	83.1	82.4	79.2	81.7	0.668
≥ Five years’ mining experience	38.9	56.3	41.1	49.7	0.001***
Older than 35 years old	42.9	47.8	33.6	43.4	0.002**
Married	37.3	47.2	32.8	42.0	0.005**
Secondary education	24.7	34.4	27.2	31.1	0.079
Had a prior episode of malaria in the past year	68.3	67.8	62.4	66.5	0.361
Owns mobile phone	61.8	77.8	61.7	71.2	< 0.001***
Access to mass media channels	44	53.4	38.2	48.1	0.019*
Listens to the radio frequently	8.7	16.2	13	14.2	0.168
Watches TV frequently	25.5	21.3	15.8	20.6	0.333
Access internet frequently	22.7	35	15.9	28.2	< 0.001***
Access social media frequently	18.7	31.8	16.5	25.9	< 0.001***

*p < 0.5, **p < 0.01, ***p < 0.05

Table 3 highlights the prevalence of malaria care-seeking and treatment ideation constructs among miners with a history of fever in Guyana. The majority (92%) of miners had high perceived severity of malaria, while two-thirds had high levels of perceived self-efficacy to protect themselves from malaria (67%). Over one-half of miners had high knowledge of (57%) and perceived susceptibility to malaria (53%), while 49% had high levels of belief in the efficacy of testing and treatment for malaria protection and positive beliefs about malaria, respectively. On the other hand, only about one-fifth of miners either perceived social norms that support treatment adherence (21%) or engaged in interpersonal communication regarding malaria prevention (20%). Miners in Region 7 tended to have significantly higher levels of perceived severity, susceptibility, norms, response efficacy, and positive beliefs regarding malaria care-seeking or treatment compared to miners in Region 1 or 8. No significant regional differences were observed regarding knowledge, perceived self-efficacy, or interpersonal communication.

**Table 3:** Percent of respondents with positive ideation constructs, by region

Ideation constructs	Prevalence				Chi-square
	Region 1 (n = 157)	Region 7 (n = 386)	Region 8 (n = 202)	Total (n = 745)	P-value
High knowledge	58.5	59.3	51.7	57.3	0.319
Perceived severity	88.3	95.6	86.7	92.2	0.003**
Perceived susceptibility	62.4	55.2	41.4	52.9	0.006**
Perceived norms	19.8	23.9	12.9	20.5	0.007**
Perceived self-efficacy	65	67	69.7	67.3	0.765
Perceived response efficacy	42.5	55.2	37.8	48.8	< 0.001***
Positive beliefs	38.9	52.5	48.6	49.4	0.034*
Advocacy/Interpersonal communication	16.4	22.7	16.3	20.1	0.127

*p < 0.5, **p < 0.01, ***p < 0.05

Table 4 highlights mean ideation scores by miners’ characteristics. Overall, miners' care-seeking/treatment ideation score ranged from 0 to 8 with a mean of 4.1 (standard error (SE) = 0.1). Fifty-nine percent of miners had a low ideation score (< 5), while 41% had a high ideation score (≥ 5 or more). Mean ideation scores were significantly higher among miners who were in Region 7, older than 35 years, more educated, owned a mobile phone, and with access to mass media channels.

**Table 4:** Mean ideation score and proportion of miners with high and low scores, by characteristics of the sample

	Mean (standard error [SE]) Ideation Score (n = 745)	% Low Ideation (score < 5) n = 454	% High Ideation (score ≥ 5) n = 291	P-value
Overall	4.1 (0.1)	59.3%	40.7%	n/a
Region				
1	3.9 (0.2)	64.4	35.6	0.002
7	4.3 (0.1)	53.2	46.8	
8	3.7 (0.1)	70.2	29.8	
Sex				
Female	4.1 (0.1)	63.5	36.5	0.437
Male	4.1 (0.1)	58.6	41.4	
Religion				
Other	3.9 (0.3)	61	39	0.743
Christian	4.1 (0.1)	58.9	41.1	
Age				
≤ 35 years	3.9 (0.1)	64.3	35.7	0.004
> 35 years	4.3 (0.1)	52.7	47.3	
Marital status				
Not married	4.0 (0.1)	59.3	40.7	0.992
Married	4.2 (0.1)	59.2	40.8	
Education				
< Secondary	4.0 (0.1)	60.3	39.7	0.566
≥ Secondary	4.3 (0.1)	57	43	
Mining experience				
< 5 years	4.0 (0.1)	61.6	38.4	0.289
5 or more	4.2 (0.1)	56.9	43.1	
Prior episode of confirmed malaria				
No	3.9 (0.1)	63.3	36.7	0.154
Yes	4.2 (0.1)	57.2	42.8	
Owns mobile phone				
No	3.6 (0.1)	72.9	27.1	< 0.001
Yes	4.3 (0.1)	53.8	46.2	
Accesses media channels frequently				
No	3.8 (0.1)	66.9	33.1	< 0.001
Yes	4.4 (0.1)	51	49	

Table 5 summarizes the relationships between overall ideation score and the behavioural outcomes of interest (any care-seeking, prompt care-seeking, getting tested for malaria, and self-treatment), as well as the relationships between individual ideation variables and those same behavioural outcomes. Miners’ ideation was significantly associated with care-seeking and treatment outcomes. Specifically, each unit increase in miners’ ideation scores resulted in significantly higher odds of care-seeking for fever (adjusted odds ratio [aOR]: 1.19; 95% confidence interval [CI] 1.04–1.36), getting tested for malaria (aOR: 1.22; 95% CI 1.07–1.38) and lower odds of self-medication for their symptoms (aOR: 0.87; 95% CI 0.77–0.99).

**Table 5:** Associations between miner’s ideation and malaria care-seeking, testing, and self-medication

Overall ideation	aOR (95% CI) of care-seeking and treatment outcomes			
	Any care-seeking	Prompt care-seeking	Testing	Self-medication
Ideation score (per 1 unit increase)	1.19 (1.04–1.36)	1.10 (0.93–1.30)	1.22 (1.07–1.38)	0.87 (0.77–0.99)
Ideation variables				
Knowledge	1.56 (1.10–2.20)	1.39 (0.97–1.99)	1.63 (1.17–2.29)	0.98 (0.71–1.34)
Perceived severity	0.83 (0.40–1.74)	0.98 (0.46–2.09)	0.67 (0.34–1.34)	0.74 (0.38–1.46)
Perceived susceptibility	0.98 (0.70–1.37)	1.36 (0.95–1.96)	0.95 (0.54–1.68)	1.00 (0.70–1.42)
Perceived norms	1.28 (0.80–2.05)	1.09 (0.68–1.75)	1.53 (1.07–2.19)	0.86 (0.55–1.34)
Perceived self-efficacy	1.25 (0.88–1.79)	1.02 (0.71–1.45)	1.51 (1.01–2.27)	0.74 (0.51–1.07)
Perceived response efficacy	1.51 (1.02–2.24)	1.24 (0.86–1.78)	1.41 (1.02–1.95)	0.70 (0.51–0.95)
Beliefs	1.30 (0.94–1.79)	1.01 (0.68–1.49)	1.32 (0.98–1.80)	0.66 (0.46–0.94)
Advocacy/Interpersonal communication	1.41 (0.90–2.22)	0.99 (0.62–1.56)	1.34 (0.75–2.39)	0.99 (0.64–1.54)

Adjusted for region, sex, religion, age, marital status, education, mining experience, prior episode of malaria, ownership of a mobile phone and access to mass media channels

However, ideation scores were not significantly associated with prompt care-seeking. Specific ideation variables associated with care-seeking include knowledge (aOR: 1.56; 95% CI 1.10–2.20) and perceived response efficacy (aOR: 1.51; 95% CI 1.02–2.24), while prompt care-seeking was associated with knowledge (aOR: 1.39; 95% CI 0.97–1.99) and perceived susceptibility (aOR: 1.36; 95% CI 0.95–1.96). Malaria testing was influenced by miners’ knowledge (aOR: 1.63; 95% CI 1.17–2.29), perceived norms (aOR: 1.53; 95% CI 1.07–2.19), perceived self-efficacy (aOR: 1.51; 95% CI 1.01–2.27), perceived response efficacy (aOR: 1.41; 95% CI 1.02–1.95) and positive beliefs (aOR: 1.32; 95% CI 0.98–1.80). In addition, miners with perceived response efficacy (aOR: 0.70; 95% CI 0.51–0.95) and positive beliefs (aOR: 0.66; 95% CI 0.46–0.94) were less likely to self-medicate.

**Fig. 1:**
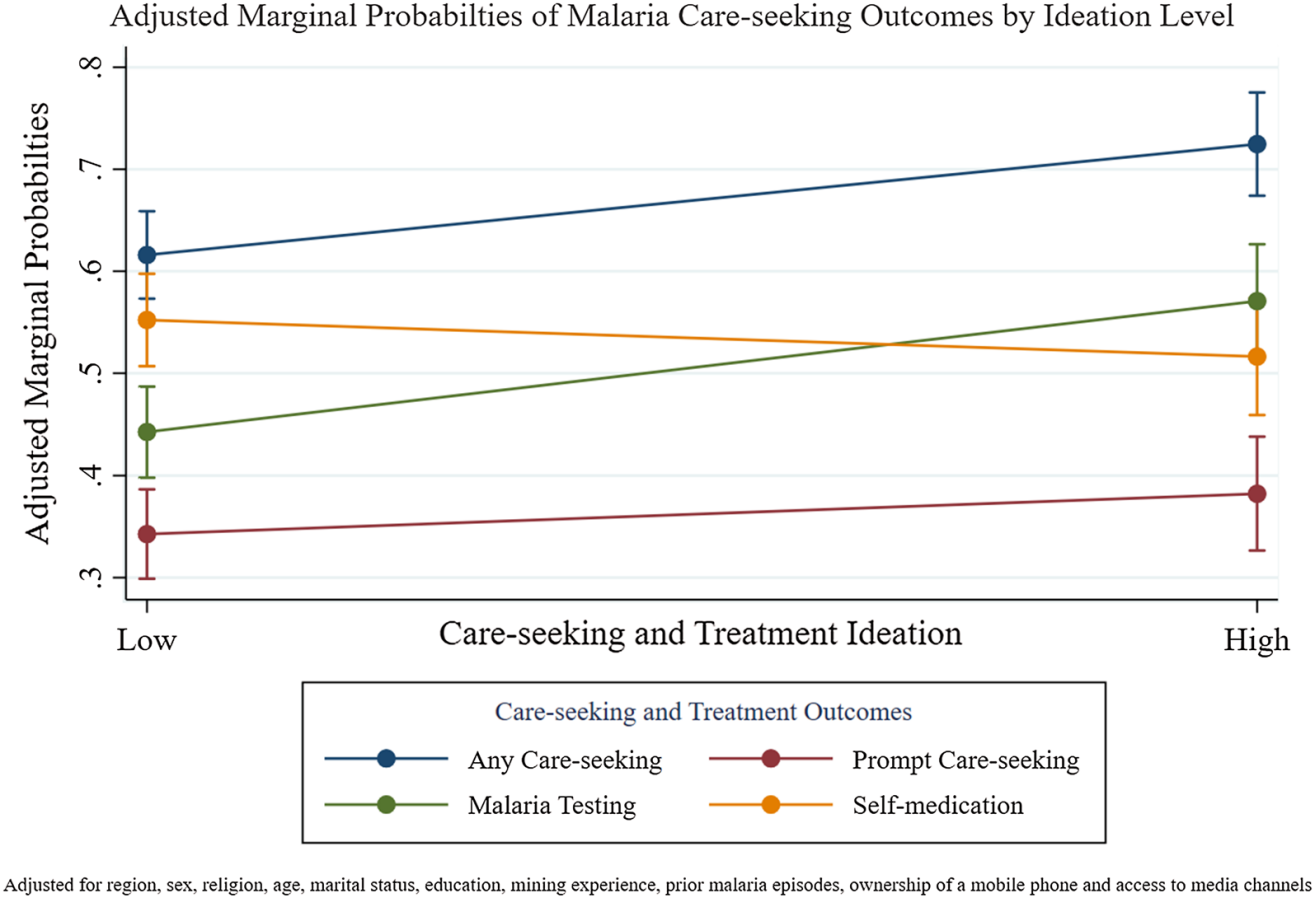
Adjusted marginal probabilities of malaria care-seeking outcomes by ideation level. Adjusted for region, sex, religion, marital status, education, mining experience, prior malaria episodes, ownership of a mobile phone and access

As shown in Fig. 1, miners with a fever in the last 12 months with high ideation had higher adjusted marginal probabilities of seeking any care (11 percentage points (pp) increase), prompt care-seeking (4 pp increase), malaria testing (13 pp increase) and lower adjusted marginal probability of self-medication (4 pp decrease) compared to those with low levels of ideation.

## Discussion

This is the first study in Guyana to explore care-seeking ideation among the gold mining population. This study is the most comprehensive one to date on the complex dynamics of behavioural decision-making related to malaria among populations in remote regions of Guyana most susceptible to the disease. It used an integrated conceptual model, known as ideation, to better understand decision-making about care-seeking, testing, and use of approved treatments for malaria prevention and mitigation. Studies show that multiple psychosocial, social, contextual, and structural factors can affect malaria-related behaviour, the ideational approach sheds light on how multiple factors often interact and have simultaneous influence over healthcare decisions. This allows for more nuanced and sophisticated communication strategies that are more likely to be effective for particular behaviours, audiences, and localized contexts.

Several common theories of behaviour change recognize that perceptions of threat and efficacy can be motivating; people who recognize that they are at risk and either have confidence in themselves and in available services or response options are more likely to be proactive in exercising healthy behaviours to address the disease threat. Also, living in a social environment where behavioural norms support protective actions and where people talk about the health issues they face and how to deal with them, can be reinforcing factors. This study found that miners generally have relatively high perceptions of malaria risk and susceptibility, as well as relatively high knowledge about malarial disease, high self-confidence (self-efficacy) to protect themselves, and some trust in available testing and treatment options (response efficacy). This would normally indicate favourable conditions for the exercise of positive care-seeking, malaria testing, and acceptance of approved treatments. On the other hand, this study also found that miners in Guyana had relatively low perceptions of normative support for these protective behaviours and reported relatively little communication with others about these issues. That suggests relatively weak social support to engage in these key protective behaviours, which could reduce otherwise positive motivation to act [16]. This might be explained by the fact that miners are transitory and may have reduced chances of forming strong communities. Qualitative studies have also shown that self-medication among miners is borne out of conveniences as well as the belief that self-treatment works [21].

The advantage of the ideational analysis approach is that, while it allows inspection of the individual variables that go into a cumulative ideation score, it also emphasizes analysis of all of these factors simultaneously—recognizing the potential for interactions among them—as they apply to each of the three outcome behaviours of interest, while also controlling for the various demographic and contextual variables that define the local environments of miners in different regions and communities. When the cumulative ideation scores were calculated for each of the three outcome behaviours, the study found relatively low care-seeking and treatment ideation, which normally would be linked to a lower probability of those behaviours. However, the ideation score varied across individuals and subgroups; it was higher among miners in Region 7 and among older, more educated, and more digitally connected miners. Other studies exploring ideation among key populations such as pregnant women and children also reported variations in ideation scores across and within subgroups [18, 19].

Miners who had higher care-seeking and testing ideation scores—indicating the presence of more positive ideational factors—were more likely to report those behaviours and were less likely to report self-medication rather than using approved treatment regimens. While certain individual ideational factors were more strongly associated with some outcomes than others (e.g., perceived normative support correlated with greater odds of getting tested, but not with care-seeking or rejecting self-medication), the cumulative ideation score was associated with greater adjusted marginal odds for each of the outcome behaviours. This suggests important programmatic value in communication strategies that emphasize multiple factors, rather than single factors, especially among miners with lower ideation to begin with. Most notably, this includes younger miners, those with less education, those with less access to mobile phones and other media, and those in Regions 1 and 8.

For example, according to this study, care-seeking messages should focus on increasing miners’ knowledge of malaria transmission and symptoms, as well as the conditions in the mining camps that make a miner susceptible to the disease. In contrast, messages about malaria testing should combine information about malaria knowledge, with encouragement of positive beliefs about malaria testing and volunteer testers, evidence about the effectiveness of testing as a protective strategy, reminders of how quick and easy it is to get a malaria test somewhere nearby, and the fact that one’s peers will support you if you get tested when you have a fever. This analysis can inform both media messaging as well as outreach efforts in remote regions, where mobile health workers can be guided with talking points about multiple factors to emphasize when discussing particular behaviours with their clients.

The study findings are being used in the implementation of SBC interventions for miners. The NMP and Breakthrough ACTION Guyana project are implementing a mass media campaign informed by human-centred design to address miners’ ideation related to care-seeking and treatment and increase demand for malaria related services [22]. The campaign spans a variety of channels including radio, television, print materials, and social media. Central to the campaign is the use of a fictional miner’s experience and messages to increase risk perception, malaria knowledge, prompt care-seeking, and treatment adherence. This is accompanied by other influential persons in the mining community speaking out on the need for prompt and proper care-seeking for malaria. The mass media campaign is complemented by other interventions to ensure the delivery of high quality of testing and treatment services as the CCM initiative is being scaled up by the NMP. The Breakthrough ACTION Guyana project is complementing the NMP efforts by branding malaria testing and treatment locations with flags to increase the visibility of the CCM programme. The volunteer testers are given certificates after training to promote their buy-in as well as validate them within their communities. The volunteer testers are also given job aids such as rapid counselling cards and a treatment pocket guide to improve the quality and accuracy of the service provided. Treatment adherence handouts are given to malaria positive clients that illustrate how the treatment works to encourage treatment adherence. An endline survey of miners is to be conducted to assess the impact of these interventions on miners’ behaviours and malaria outcomes.

While SBC interventions have been shown to improve miners' behaviours and potentially impact malaria outcomes [23], context-specific structural solutions remain crucial to the elimination of malaria in Guyana. The inaccessibility of hinterland regions, cost needed to reach the regions, the fact that houses in the hinterland are not conducive to indoor spraying and the emergence of insecticide resistance, has made the use of larvicides and indoor residual spraying unsustainable [24, 25]. Additionally, in-country migration due to humanitarian crises across Guyana’s borders have also contributed to the continuous transmission and endemicity of malaria. This study also has implications for efforts to eliminate malaria across the Guiana Shield, including Suriname [26], Brazil [24] and Venezuela [27], which have been also impeded by gold mining, high rates of self-medication due to inaccessible health facilities, and the importation of malaria cases [28]. Efforts to ensure sustainability of programmatic interventions must be thoroughly explored. The role of public–private partnerships has proven to be a promising option [29].

This study has some limitations. First, the study relies wholly on self-reported data from miners, which may be prone to recall or social desirability bias. Only miners who were present in mining camps on the day of data collection were interviewed. The cross-sectional study design limits the ability to infer causality from the associations observed. The study was unable to explore treatment adherence among miners who were given malaria treatment due to low sample size and insufficient power. Also, the study did not explore supply side factors occurring at the malaria service provision sites, which may likely influence miners’ behaviours. Such factors may include stockout of RDTs and anti-malarial drugs and inadequate technical and interpersonal skills of the service providers.

## Conclusion

The National Malaria Program is using study findings during the scale-up of its community case management initiative, using volunteers to make malaria testing and treatment services more accessible to miners. Most miners with a recent episode of fever had high levels of care-seeking and treatment-related perceived risk, self-efficacy, susceptibility, and knowledge. Miners' care-seeking/treatment ideation varied across subgroups and ideation scores were associated with higher odds of care-seeking for fever, getting tested for malaria, and lower odds of self-medication. The NMP is using these study findings during the scale up of its CCM initiative, using volunteers to make testing and treatment services more accessible to miners. This is complemented by a multi-channel mass media campaign to improve miners’ ideation. Study findings also have implications for efforts to eliminate malaria across the Guiana Shield.

## Data Availability

The datasets used and analysed during the current study are available from the corresponding author on reasonable request.

## Abbreviations

aOR: Adjusted odds ratio
CCM: Community case management
CI: Confidence interval
NMP: National Malaria Programme
RDT: Rapid diagnostic test
SBC: Social and behaviour change
SE: Standard error
